# Biography

**Published:** 1919-08

**Authors:** 


### BIOGRAPHY

---

DR. JAMES McMANUS.—Dr. McManus, on July 16, celebrated his eighty-third birthday by sending a message of good cheer to his numerous professional friends. Few men have been as loyal and faithful in the service of so extended a professional career. He has not only been a faithful servant of the patients who have confided to his care their most important and vital interest, but he has also been a faithful and devoted member of an honorable profession. That he may live many more years, to witness future triumphs of his beloved profession, is the cordial and unanimous wish of his admiring professional confreres.

---

DR. JOHN C. McCOY.—The *Pacific Gazette* of July records the death of Dr. McCoy and makes a most admirable tribute to the works of a good and faithful professional man. Dr. McCoy was not widely known for the things that make men famous, but he made a most honorable and faithful contribution to the profession in his adopted state, and his patients and friends will remember him with sincere gratitude. He was a great man and died too soon; but his life should stimulate others to emulate higher things.

